# Supplementary material for: Insensitive High-Energy Density Materials Based on Azazole-Rich Rings: 1,2,4-Triazole N-Oxide Derivatives Containing Isomerized Nitro and Amino Groups
Source: Int J Mol Sci. 2023 Feb 15;24(4):3918. doi: 10.3390/ijms24043918 (PMC9962610; doi:10.3390/ijms24043918)
Supplement: Supplementary file 1 [file ijms-24-03918-s001.zip › ijms-2187106-supplementary.pdf]

Supplementary Materials

Insensitive High-Energy Density Materials Based on Azazole-Rich Rings: 1,2,4-Triazole *N*-Oxide Derivatives Containing Isomerized Nitro and Amino Groups

Xinbo Yang [1,2], Nan Li [1], Yuchuan Li [2,*] and Siping Pang [2,*]

[1] School of Mechatronical Engineering, Beijing Institute of Technology, Beijing 100081, China
[2] School of Materials Science and Engineering, Beijing Institute of Technology, Beijing 100081, China
* Correspondence: liyuchuan@bit.edu.cn (Y.L.); pangsp@bit.edu.cn (S.P.)

Table of contents

1. Table S1. The bond length (Å) and Mayer bond order (MBO) of the NATNOs molecules at M06-2X/6-311G (d, p). 3
2. Table S2. Calculated bond dissociation enthalpy (BDE, kJ/mol) and Mayer bond order (MBO) of trigger bond for designed NATNOs and other energetic compounds. 5
3. Figure S1. Standard coloring method and chemical explanation of $sign(\lambda_2)\rho$ on IRI isosurfaces. 6
4. References 7

Table S1. The bond length (Å) and Mayer bond order (MBO) of the NATNOs molecules at M06-2X/6-311G (d, p) [1,2].

NATNO-1

Bond	Length /(Å)	MBO	Bond	Length /(Å)	MBO
C1-N9	1.388	0.977	N7-O8	1.243	1.369
C2-N9	1.331	1.234	N9-O10	1.289	1.182
C2-N6	1.356	1.029	N14-O15	1.205	1.546
N6-N7	1.393	0.952	N14-O16	1.209	1.521
C1-N7	1.329	1.171	N11-H12	1.019	0.909
C1-N14	1.438	0.761	N11-H13	1.016	0.908
C2-N3	1.349	1.159	N3-H4	1.016	0.884
N6-N11	1.369	1.033	N3-H5	1.009	0.910

NATNO-2

Bond	Length /(Å)	MBO	Bond	Length /(Å)	MBO
C1-N3	1.370	1.031	N14-O15	1.222	1.415
C2-N3	1.340	1.181	N11-O12	1.226	1.414
C2-N4	1.311	1.474	N11-O13	1.200	1.569
N4-N14	1.353	1.118	N8-H9	1.018	0.874
C1-N14	1.364	1.044	N8-H10	1.016	0.883
C1-N11	1.422	0.809	N5-O6	1.218	1.453
N3-N8	1.403	0.945	N5-O7	1.196	1.583
C2-N5	1.455	0.742			

NATNO-3

Bond	Length /(Å)	MBO	Bond	Length /(Å)	MBO
C1-N14	1.367	1.091	N10-N11	1.390	0.953
C2-N14	1.384	1.046	N7-O8	1.200	1.572
C2-N3	1.321	1.425	N7-O9	1.221	1.448
N3-N10	1.301	1.274	N4-O5	1.205	1.548
C1-N10	1.352	1.021	N4-O6	1.208	1.515
C1-N7	1.427	0.793	N11-H12	1.017	0.884
N14-O15	1.239	1.354	N11-H13	1.016	0.907
C2-N4	1.447	0.762			

NATNO-4

Bond	Length /(Å)	MBO	Bond	Length /(Å)	MBO
C1-N13	1.292	1.606	N9-N10	1.37090	1.01764357
C2-N13	1.338	1.346	N6-O7	1.20012	1.55769196
C2-N14	1.352	1.120	N6-O8	1.21073	1.51009315
N9-N14	1.386	0.990	N3-O4	1.20480	1.53544732
C1-N9	1.354	1.073	N3-O5	1.20775	1.52487003
C1-N6	1.459	0.722	N10-H11	1.01704	0.89977255
C2-N3	1.450	0.741	N10-H12	1.02044	0.89726368
N14-O15	1.230	1.409			

NATNO-5

Bond	Length /(Å)	MBO	Bond	Length /(Å)	MBO
C1-N3	1.36023	1.09181720	N15-N16	1.36736	1.05495794
C2-N3	1.38912	0.95167668	N10-O11	1.25262	1.28031697
C2-N13	1.32916	1.17383100	N10-O12	1.24622	1.32850288
N13-N15	1.42129	0.90458959	N4-H5	1.02266	0.87229998
C1-N15	1.37904	0.94925981	N4-H6	1.01760	0.89251523
C1-N10	1.36148	1.01010015	N7-O8	1.21901	1.45977501
N3-N4	1.42440	0.92148490	N7-O9	1.20499	1.53347786
C2-N7	1.42416	0.81508806	N16-H17	1.01628	0.91715597
N13-O14	1.20874	1.50416145	N16-H18	1.01373	0.90027569

NATNO-6

Bond	Length /(Å)	MBO	Bond	Length /(Å)	MBO
C1-N15	1.35809	1.10029359	N12-N17	1.38577	0.99301203
C2-N15	1.39716	1.01925156	N6-O7	1.21052	1.51027115
C2-N18	1.37186	0.97363786	N6-O8	1.21451	1.49811397
N17-N18	1.43819	0.88137217	N3-O4	1.20980	1.51040375
C1-N17	1.39032	0.96389397	N3-O5	1.21567	1.48985099
C1-N6	1.42589	0.82494238	N9-H10	1.01214	0.90717164
N15-O16	1.23119	1.38518564	N9-H11	1.01481	0.91362520
C2-N3	1.43273	0.85381793	N12-H13	1.01654	0.91002210
N9-N18	1.36454	1.03407578	N12-H14	1.00882	0.91345898

Table S2. Calculated bond dissociation enthalpy (BDE, kJ/mol) and Mayer bond order (MBO) of trigger bond for designed NATNOs and other energetic compounds.

Compound	Trigger Bond	MBO	BDE [a]/kJ/mol
IHEM-1	N-NO_2	0.8643	313.36 (290.49 [3])
ANTA	C-NO_2	0.7194	299.16 (294.14 [4])
HDNT	C-NO_2	0.7193	286.00
**NATNO**	N-NO_2	0.8020	232.38
**NATNO-1**	C-NO_2 (C1-N14)	0.7610	173.60
	N-NH_2 (N6-N11)	1.0335	111.37
**NATNO-2**	C-NO_2 (C1-N11)	0.8093	199.58
	C-NO_2 (C2-N5)	0.7423	270.71
	N-NH_2 (N3-N8)	0.9451	181.42
**NATNO-3**	C-NO_2 (C1-N7)	0.7933	201.34
	C-NO_2 (C2-N4)	0.7617	264.53
	N-NH_2 (N10-N11)	0.9530	221.42
**NATNO-4**	C-NO_2 (C1-N6)	0.7220	262.34
	C-NO_2 (C2-N3)	0.7412	284.05
	N-NH_2 (N9-N10)	1.0176	204.27
**NATNO-5**	C-NO_2 (C2-N7)	0.8151	62.61
	N-NH_2 (N3-N4)	0.9215	-0.74
	ring (N13-N15)	0.9046	20.82
**NATNO-6**	C-NO_2 (C1-N6)	0.8249	47.97
	C-NO_2 (C2-N3)	0.8538	47.97
	ring (N17-N18)	0.8814	8.90
NTO	C-NO_2	0.7492	292.36 (276.00 [5])
TATB	C-NO_2	0.8454	305.40 (355.00 [6])
FOX-7	C-NO_2	0.8253	298.59 (280.33 [7])
RDX	N-NO_2	0.8824	182.48 (211.66 [8])
HMX	N-NO_2	0.9486	179.86 (194.56 [9])
CL-20	N-NO_2	0.8689	171.96 (186.61 [10])

[a] BDEs in the table were calculated at the RI-PWPB95-D3(BJ)/def2-QZVPP//M06-2X/6-311G (d, p) theoretical level. The values in parentheses are literature values.

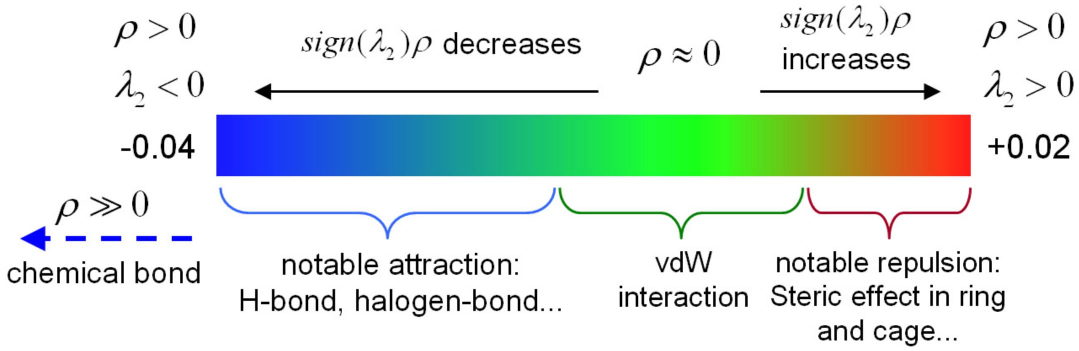

Figure S1. Standard coloring method and chemical explanation of $sign(\lambda_2)\rho$ on IRI isosurfaces [11].

References

1. Zhao, Y.; Truhlar, D.G. The M06 Suite of Density Functionals for Main Group Thermochemistry, Thermochemical Kinetics, Noncovalent Interactions, Excited States, and Transition Elements: Two New Functionals and Systematic Testing of Four M06-class Functionals and 12 other Functionals. *Theor. Chem. Acc.* **2008**, *120*, 215–241.
2. Wong, M.W.; Gill, P.M.W.; Nobes, R.H.; Radom, L. 6-311G(MC)(d,p): A Second-row Analogue of the 6-311G(d,p) Basis Set: Calculated Heats of Formation for Second-row Hydrides. *J. Phys. Chem.* **1988**, *92*, 4875–4880.
3. Zhang, J.; Feng, Y.; Bo, Y.; Staples, R.J.; Zhang, J.; Shreeve, J.M. One Step Closer to an Ideal Insensitive Energetic Molecule: 3,5-Diamino-6-hydroxy-2-oxide-4-nitropyrimidone and its Derivatives. *J. Am. Chem. Soc.* **2021**, *143*, 12665–12674.
4. Sorescu, D.C.; Bennett, C.M.; Thompson, D.L. Theoretical Studies of the Structure, Tautomerism, and Vibrational Spectra of 3-Amino-5-nitro-1,2,4-triazole. J. Phys. Chem. A. 1998, 102, 10348–10357.
5. Türker, L.; Bayar, Ç.Ç. NTO-Picryl Constitutional Isomers-A DFT Study. J. Energ. Mater. 2011, 30, 72–96.
6. Tang, Y.; Zhang, J.; Mitchell, L.A.; Parrish, D.A.; Shreeve, J.M. Taming of 3,4-Di(nitramino)furazan. J. Am. Chem. Soc. 2015, 137, 15984–15987.
7. Jiang, H.; Jiao, Q.; Zhang, C. Early Events When Heating 1,1-Diamino-2,2-dinitroethylene: Self-Consistent Charge Density-Functional Tight-Binding Molecular Dynamics Simulations. J. Phys. Chem. C. 2018, 122, 15125–15132.
8. Wang, Q.; Shao, Y.; Lu, M. C8N12O8: A Promising Insensitive High-Energy-Density Material. Cryst. Growth Des. 2018, 18, 6150–6154.
9. Liu, G.; Tian, B.; Wei, S.-H.; Zhang, C. Polymorph-Dependent Initial Thermal Decay Mechanism of Energetic Materials: A Case of 1,3,5,7-Tetranitro-1,3,5,7-Tetrazocane. J. Phys. Chem. C. 2021, 125, 10057–10067.
10. Kiselev, V.G.; Goldsmith, C.F. Accurate Prediction of Bond Dissociation Energies and Barrier Heights for High-Energy Caged Nitro and Nitroamino Compounds Using a Coupled Cluster Theory. J. Phys. Chem. A. 2019, 123, 4883–4890.
11. Lu, T.; Chen, Q. Interaction Region Indicator: A Simple Real Space Function Clearly Revealing Both Chemical Bonds and Weak Interactions**. Chem–Methods 2021, 1, 231–239.
